# The Influence of EGFR Inactivation on the Radiation Response in High Grade Glioma

**DOI:** 10.3390/ijms19010229

**Published:** 2018-01-12

**Authors:** Oana Alexandru, Stefana Oana Purcaru, Ligia Gabriela Tataranu, Laura Lucan, Juan Castro, Catalin Folcuţi, Stefan-Alexandru Artene, Cristian Tuţă, Anica Dricu

**Affiliations:** 1 Faculty of Medicine, University of Medicine and Pharmacy of Craiova, Str. Petru Rares nr. 2-4, 710204 Craiova, Romania; oanale@hotmail.com (O.A.); stoapo@yahoo.com (S.O.P.); lauralucan29@gmail.com (L.L.); catalin_folcuti@yahoo.com (C.F.); stefan.artene@yahoo.com (S.-A.A.); tutacristi@yahoo.com (C.T.); 2 Department of Neurosurgery, “Bagdasar-Arseni” Emergency Hospital, Șoseaua Berceni 12, 041915 Bucharest, Romania; ttranu@gmail.com; 3 Karolinska Institutet, Department of Oncology-Pathology, Cancer Center Karolinska, Karolinska University Hospital, Z1:00, 171 76 Stockholm, Sweden; Juan.Castro@ki.se

**Keywords:** high grade glioma (HGG), radiotherapy, epidermal growth factor receptor (EGFR)

## Abstract

Lack of effectiveness of radiation therapy may arise from different factors such as radiation induced receptor tyrosine kinase activation and cell repopulation; cell capability to repair radiation induced DNA damage; high grade glioma (HGG) tumous heterogeneity, etc. In this study, we analyzed the potential of targeting epidermal growth factor receptor (EGFR) in inducing radiosensitivity in two human HGG cell lines (11 and 15) that displayed similar growth patterns and expressed the receptor protein at the cell surface. We found that 15 HGG cells that express more EGFR at the cell surface were more sensitive to AG556 (an EGFR inhibitor), compared to 11 HGG cells. Although in line 15 the effect of the inhibitor was greater than in line 11, it should be noted that the efficacy of this small-molecule EGFR inhibitor as monotherapy in both cell lines has been modest, at best. Our data showed a slight difference in the response to radiation of the HGG cell lines, three days after the treatment, with line 15 responding better than line 11. However, both cell lines responded to ionizing radiation in the same way, seven days after irradiation. EGFR inhibition induced radiosensitivity in 11 HGG cells, while, in 15 HGG cells, the effect of AG556 treatment on radiation response was almost nonexistent.

## 1. Introduction

The clinical oncology landscape has changed dramatically in the last decades with the introduction of targeted therapy and, more recently, to the addition of immunotherapy. As more and more molecular agents are steadily inducted into clinical protocols, patients who previously could only benefit from chemotherapy and radiotherapy, can now receive two, three even four lines of treatment before the exhaustion of all therapeutic options.

Malignant gliomas are a family of brain tumors originated from glial cells. A distinct group of malignant gliomas, called astrocytomas are derived from astrocytes and are the most numerous and the most aggressive malignancies of the Central Nervous System (CNS). Astrocytomas have been classified by the World Health Organization (WHO) on the basis of hystology and prognosis into two categories: low grade gliomas (LGG) and high grade gliomas (HGG). LGG are comprised of grade I (pylocitic astrocytomas) and grade II (diffuse astrocytomas) and generally slow-progressing tumors with an overall good prognosis. HGG are comprised of grade III (anaplastic astrocytomas) and grade IV (glioblastomas) (GBMs), which are both the most numerous and the most aggressive tumors of the CNS, being considered incurable in 99% of the cases. Despite the groundbreaking progress made, treatment for some malignancies, such as GBMs, has remained almost completely unchanged for the better part of the last 10 years. 

The protocol involving surgery and adjuvant hyperfractionated radiotherapy alongside the alkylating agent Temozolomide (TMZ) [1], has remained the standard of care for patients suffering from GBMs, with a median overall survival of 14.6 months that is not sufficient to improve the abysmal outcome from GBM patients. Insulin like growth factor-1 receptor (IGF-1R), Platelet-derived growth factor receptor (PDGFR), vascular endothelial growth factor receptor (VEGFR), tyrosine-kinase receptor encoded by the KIT locus (KIT), and epidermal growth factor receptor (EGFR) were reported to be overexpressed or constitutive activated by gene deletion, amplification of rearrangement, in malignant glioma [2,3,4,5,6].One of the most popular treatments which was intensely trialed during this period for GBMs involved the Epithelial Growth Factor (EGF) and its’s receptor, the Epithelial Growth Factor Receptor (EGFR). EGF/EGFR inhibition was driven by the large role played in glioma genesis and in mechanisms involved in tumor growth, such as reduced apoptosis, proliferation, invasion or angiog1enesis. The most notable example is the presence of the EGFRvIII, the most common mutation in GBMs which is encountered in one third of all tumors [7,8] and is involved in increased progression, reduced apoptosis and hampered chemo/radiotherapy resistance [9,10], its presence being indicative for an overall worse prognosis [11]. Additionally, other EGFR alterations are present within GBMs, with almost three quarters of patients presenting EGFR amplifications in tandem with other EGFR mutations and/or other structural alterations [12,13]. While EGF/EGFR inhibition showed great potential along the years [14,15] results from phase I/II clinical trials failed to show any benefit for patients with GBM. Monoclonal antibodies such as cetuximab or nimotuzumab [16,17,18], small tyrosine kinase inhibitors such as erlotinib [19,20], lapatinib [21] and vandetinib [22] and even immunotherapic approaches such as anti-EGFR vaccination presented limited results when administered to GBM patients [23,24].

Improving the effect of radiotherapy in clinical practice, radiation type and dose may play an important role. Several other parameters are known to influence the response to radiotherapy. The presence of a small cell population, called brain tumour stem cells, was suggested to be chemo and radioresistant, inducing radio-resistance by rapid tumour repopulation after irradiation [25]. The intrinsic radioresistance of malignant cells was also reported to be an important factor in radiotherapy efficiency [26].

Interestingly, a direct link between EGFR status and radiosensibility has been shown to exist [27] with anti-EGF/EGFR molecules increasing sensibility to radiotherapy [28,29,30]. This effect has great potential given that hyperfractioned radiotherapy is still part of the standard of care for treating GBM patients.

Therefore, the main goal of our study was to increase HGG radiosensitivity through EGFR inactivation, previously reported as one of the most important targets in HGG treatment, as single*-*target or in combination with conventional therapy.

## 2. Results

### 2.1. Growth Pattern of Untreated HGG Cells

In this study, we used two cell lines (11 and 15), selected from a panel of HGG cultures, which has been previously characterized [31]. This panel of HGG cell lines was divided in 2 phenotypically distinct subsets, designated type A and type B, according to gene expression analyses and tyrosine kinase inhibitor sensitivity profile [32]. Growth rate evaluation of this cell culture panel disclosed different pattern in cell proliferation dynamic. [31], the cell lines 11 and 15 belonging to subtype B that expressed high level of mesenchymal markers and showed moderate growth proliferation, during 4 days [32]. 

**Figure 1:**
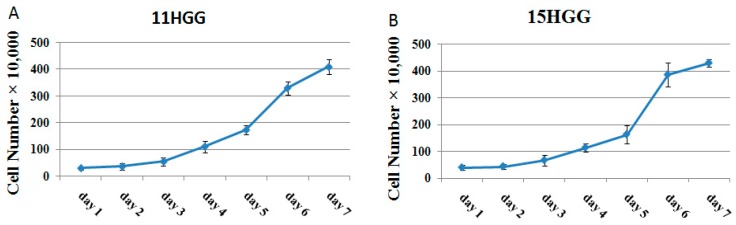
Growth curve of high grade glioma (HGG) cells. Cells were seeded into 12-well plates at a concentration of 2–5 × 10^4^ cells/well and incubated at 37 °C in standard medium. The number of the cells was determined every day by hemocytometric counting, using trypan blue. Each experiment was repeated three times. Doubling time was calculated using the slope of the logarithmic phase of growth curve.

In this study, we analyzed the proliferation dynamic of 11 and 15 HGG cell lines for a seven-day period. To generate growth curves of cell lines, untreated monolayer cells were grown on tissue culture flasks in standard culture medium for seven days; the cell growth patterns are depicted in Figure 1. Assessment of cell numbers over seven days showed that the doubling time of 11 HGG cells was 45.5 ± 5.92 h (Figure 1A) and of 15 HGG cells was 48.8 ± 4.63 h (Figure 1B). The difference in the cell doubling time (DT) was approximately 3 h, but the result was not statistically significant (*p* > 0.005).

### 2.2. The Effect of EGFR Inactivation on HGG Cells

**Figure 2:**
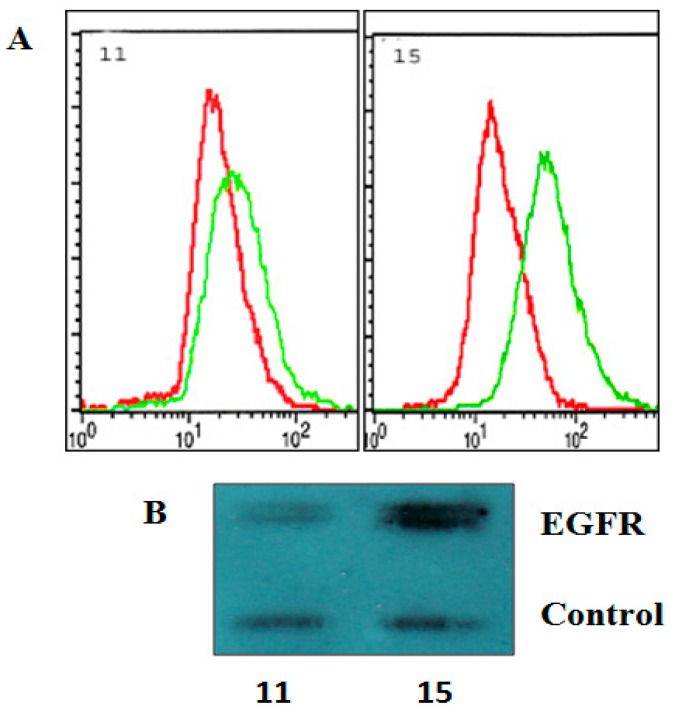
Membrane expression of EGFR on 11 HGG and 15 HGG cells. For flow cytometry determination (**A**), cells were stained with a PE-conjugated anti- EGFR, or a PE-labelled isotype Mouse IgG2B-κ control antibody (red line) and of EGFR was (green line) analyzed as described in Materials and methods; For Western blot analysis (**B**), cell lines were lysed, electrophoresed, and immunoblotted with a EGFR antibody. Membranes were reprobed with an actin antibody as a loading control.

Elevated level of wild and mutant type EGFR is a common particularity of HGG. First, we examined the level of EGFR membrane proteins in 11 and 15 HGG cells. The detection of protein receptors was made by flow cytometry (Figure 2A) and Western blotting (Figure 2B). As seen in Figure 2, both methods used in the study showed that 15 HGG cell lines expressed elevated amounts of EGFR at the cell surface, while, in 11 HGG cells, receptor levels were low (Figure 2).

EGFR blockade as monotherapy in HGG showed only modest efficacy in preclinical and clinical studies. In a study by Philip C. De Witt Hamer et al., the effect of six small molecule kinase inhibitors towards PDGFR; EGFR; mTOR; kinase insert domain receptor (KDR); fms-related tyrosine kinase 1 (FLT1) and protein kinase C beta (PKCb) were analyzed in clinical studies with GBM patients [19]. Among EGFR small molecule inhibitors existing on the market, there is limited knowledge regarding the effect of AG556 in HGG cell lines. We previously observed that AG556 has a low cytotoxic effect in several HGG cell lines [20]. To be able to draw a more accurate conclusion, we evaluated the effect of the AG556 in two more cell lines, 11 and 15 cell lines. The HGG cells were exposed to the AG556 inhibitor at concentrations of 10 μM, 20 μM and 30 μΜ. The proliferation rates were evaluated at three days and seven days respectively, as described in the Material and Methods section.

**Figure 3:**
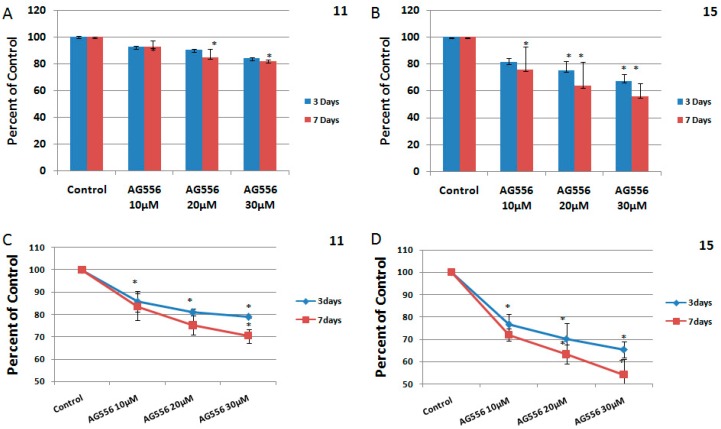
The effect of EGFR inactivation by AG556 on HGG cells. The 11 HGG (**A**,**C**) and 15 HGG (**B**,**D**) cells were seeded in 96-well culture plates or in 12-well culture plates and treated with 10 µM, 20 µM or 30 µM AG556. The cells were incubated for three or seven days and cell viability was determined by 3-(4,5-dimethylthiazol-2-yl)-2,5-diphenyl tetrazolium bromide (MTT) assay and by a Trypan Blue Exclusion Test. All results show the mean of three independent experiments ± SD, * *p* < 0.05 vs. untreated cells.

In the 11 HGG cell line, treatment with 10 μM AG556 induced 9% cytotoxicity after three days and remained unchanged after seven days (Figure 3A). Higher concentrations of AG556 (20 μM) resulted in 10% cytotoxicity in 11 HGG cells, three days after the treatment. Prolonged treatment at seven days was slightly more cytotoxic, but the result was not statistically significant (Figure 3A). We found that 30 μΜ of AG556 had the best cytotoxic effect on 11 HGG cells, reducing the survival by approximately 17% after three days but with no increase in cytotoxicity after prolonged exposure to AG 556 (seven days) (Figure 3A).

In 15 HGG cells, the treatment with AG556 reduced cell viability in a dose and time-dependent manner. Thus, three days after the administration of 10 μΜ AG556, we observed a 19% cytotoxicity while treatment with 20 μΜ induced approximately 25% cell death and 30 μΜ treatment resulted in a 33% inhibition of cell viability (Figure 3B). Seven days after the treatment, the cytotoxic effect induced by 10 μΜ AG556 was 25%. Higher concentrations of AG556 (20 μΜ) reduced cell viability by 37%, while 30 μΜ AG556 induced 44% cytotoxicity in 15 HGG cells, seven days after the treatment (Figure 3B). The effect of the AG556 treatment on HGG cells was also analyzed by a Trypan Blue Exclusion Test (Figure 3C,D). The results were not significantly different between the two methods used for determination of cell viability.

### 2.3. The Effect of Ionizing Radiation on HGG Cells

HGG resistance to radiation treatment was confirmed by several studies, both in vivo and in vitro. Our previously studies, also showed that HGG cells are resistant to ionizing radiation [26]. Here, we evaluated the effect of ionizing radiation on 11 and 15 cell lines derived from HGG. Several studies indicated that radiation induces EGFR autophosphorylation in a few minutes, which in turn causes an increase in cell proliferation [33,34]. Consequently, we considered that radiation treatment should be given concurrently with inhibition of the receptor inactivation. For this, the cells were exposed to single dose radiation of 2, 4, 6, 8 and 10 Gy of gamma radiation. The cell survival was measured by MTT assay, three and seven days after irradiation.

**Figure 4:**
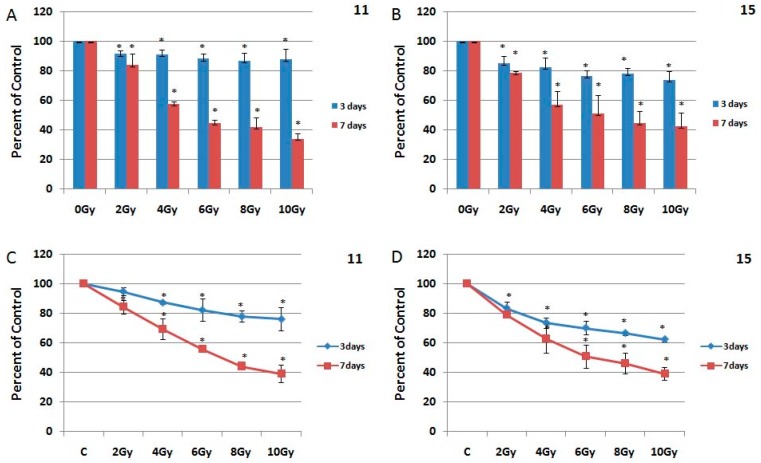
The effect of ionizing radiation on HGG cells. The 11 HGG (**A**,**C**) and 15 HGG (**B**,**D**) cells were seeded in 96-well culture plates or in 12-well culture plates and irradiated with 2 Gy, 4 Gy, 6 Gy, 8 Gy, 10 Gy and incubated for three or seven days. The cell viability was, then, determined by MTT assay and by Trypan Blue Exclusion Test. All results show the mean of three independent experiments ± SD, * *p* < 0.05 vs. untreated cells.

In 11 HGG cells, ionizing radiation caused a cytotoxic effect of 8.8% at 2 Gy, 9.3% at 4 Gy, 12% at 6 Gy, 13.4% at 8 Gy and 12.5% at 10 Gy, after 72 h. The effect of radiation was better at seven days, when the cell survival decreased with 17.2% at 2 Gy, 43.6% at 4 Gy, 55.5% at 6 Gy, 57.8% at 8 Gy and 65.9% at 10 Gy (Figure 4A).

The 15 HGG cell line proved to be more radiosensitive, three days after irradiation 2 Gy induced a cytotoxic effect of 15.1%, 4 Gy reduced cell survival by 17.7%, 6 Gy decreased cell viability by 23.8%, 8 Gy by 22.1% while 10 Gy had a cytotoxic effect of 26.5%. Seven days after the treatment, the effect of irradiation on survival of 15 HGG cells was superior, with values ranging from 21.7% at 2 Gy, 52.8% at 4 Gy, 48.8% at 6 Gy, 55.4% at 8 Gy and 67.6% at 10 Gy (Figure 4B) Compared to 11 cells, in 15 cells 2 Gy gamma radiation was 7% more cytotoxic, 4 Gy gamma radiation was 8% more cytotoxic, 6 Gy gamma radiation was 12% more cytotoxic, 8 Gy gamma radiation was 9% more cytotoxic and 10 Gy radiation was 14% more cytotoxic (Figure 4). Seven days after irradiation, 2 Gy, 4 Gy, 6 Gy, and 8 Gy ionizing radiation induced an approximately similar cytotoxic effect in both cell lines, the highest radiation dose of 10 Gy induced 8% more cytotoxic effect in 11 cells (Figure 4A,B). The effect of the gamma radiation treatment on HGG cells was also analyzed by a Trypan Blue Exclusion Test (Figure 4C,D). The results were not significantly different between the two methods used for determination of cell viability.

### 2.4. The Effect of Combined Treatment on HGG Cells Viability

Although radiotherapy is of crucial importance in HGG treatment, its effect remains problematic, due to multiple factors involved in radiation response. The combination of tumour specific agents with radiotherapy is part of a pivotal effort to increase the sensitivity of the tumor cells response to radiation.

Our next goal was to determine how EGFR inhibition induces sensitivity to radiation in the 11 and 15 HGG cell lines. For this purpose, we treated the 11 and 15 HGG cells with AG556 and concomitantly irradiated the cell lines with single doses of 2, 4, 6, 8 or 10 Gy ionizing radiation.

**Figure 5:**
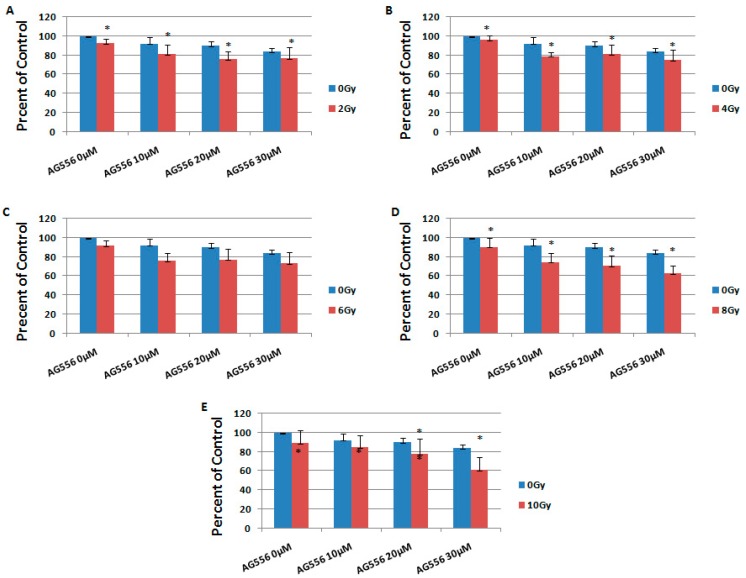
The effect of EGFR inhibition on ionizing radiation response in 11 HGG cells, three days after the treatment. The cells were seeded in 96-well culture plates (3000 cells/well) then irradiated with 2 Gy (**A**); 4 Gy (**B**); 6 Gy (**C**); 8 Gy (**D**); 10 Gy (**E**) and treated with 10 µM, 20 µM or 30 µM AG556. The cells were incubated for three days and cell viability was determined by MTT assay. All results show the mean of three independent experiments ± SD, * *p* < 0.05 vs. irradiated cells.

In the 11 cell line exposed to 2 Gy radiation, the treatment with 10 µM AG556 reduced cell survival with 12%, 20 µM AG556 treatment reduced cell survival with 17% and the treatment with 30 µM AG556 reduced cell survival with 18%, compared with radiation alone, three days after the treatment (Figure 5A).

The cytotoxic effect of 4 Gy gamma radiation was increased by 17% with the addition of 10 µM AG556, by 14% with 20 µM AG556 treatment and by 21% with 30 µM AG556 treatment, three days after the treatment (Figure 5B).

The cytotoxic effect of 6 Gy radiation was increased by 16% when using 10 µM AG556 treatment, by 15% with 20 µM AG556 treatment and by 18% with 30 µM AG556 treatment, three days after the treatment (Figure 5C).

The cytotoxic effect of 8 Gy radiation was increased by 16% after treating the cells with 10 µM AG556, by 20% with 20 µM AG556 treatment and by 28% with 30 µM AG556 treatment, three days after the treatment (Figure 5D).

The cytotoxic effect of 10 Gy ionizing radiation was increased by 4% after the addition of 10 µM AG556 treatment, by 11% after using 20 µM AG556 treatment and by 28% with 30 µM AG556 treatment, three days after the treatment (Figure 5E).

**Figure 6:**
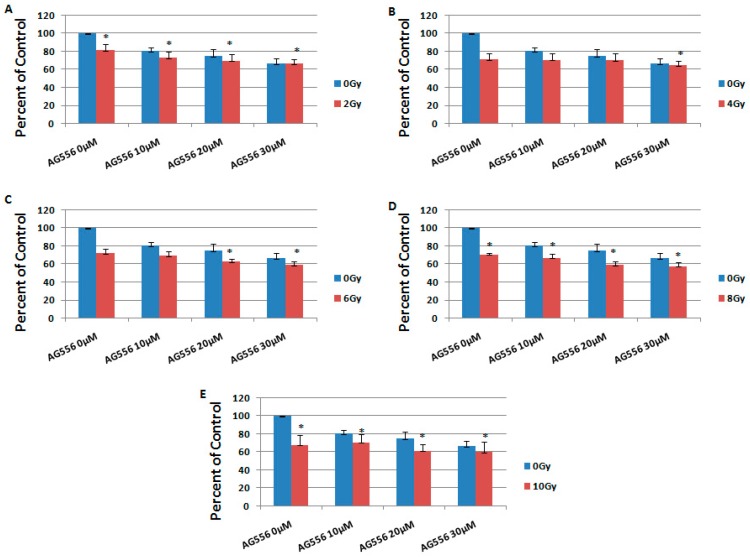
The effect of EGFR inhibition on ionizing radiation response in 15 HGG cells, three days after the treatment. The cells were seeded in 96-well culture plates (3000 cells/well) then irradiated with 2 Gy (**A**); 4 Gy (**B**); 6 Gy (**C**); 8 Gy (**D**); 10 Gy (**E**) and treated with 10 µM, 20 µM or 30 µM AG556. The cells were incubated for three days and cell viability was determined by MTT assay. All results show the mean of three independent experiments ± SD, * *p* < 0.05 vs. irradiated cells.

In 15 HGG cells irradiated with 2 Gy, the treatment with 10 µM AG556 decreased cell survival by 5%, 20 µM AG556 treatment reduced cell survival by 5% and the treatment with 30 µM AG556 reduced cell survival by 8%, compared with radiation alone, seven days after the treatment (Figure 6A).

The cytotoxic effect of 4 Gy gamma radiation was decreased by 7% with the addition of 10 µM AG556 treatment, by 1% with 20 µM AG556 treatment while 30 µM AG556 increased the effect of the combined treatment by 2%, one week after the treatment. (Figure 6B).

The cytotoxic effect of 6 Gy radiation was decreased by 7% when adding 10 µM AG556 treatment, and reduced by 3% after treatment with 20 µM AG556 and by 6% after 30 µM AG556 treatment, seven days after the treatment (Figure 6C).

The cytotoxic effect of 8 Gy radiation was decreased by 3% after treatment with 10 µM AG556, while the combined treatment with 20 µM AG556 increased the cytotoxic effect by 10% and 30 µM AG556 combined treatment reduced the cell survival by 12%, seven days after the treatment (Figure 6D).

The cytotoxic effect of 10 Gy ionizing radiation was decreased by 12% after being treated with 10 µM AG556, by 6% with 20 µM AG556 treatment, while the combined treatment with 30 µM AG556 reduced the cell survival by 3%, one week after the treatment (Figure 6E).

**Figure 7:**
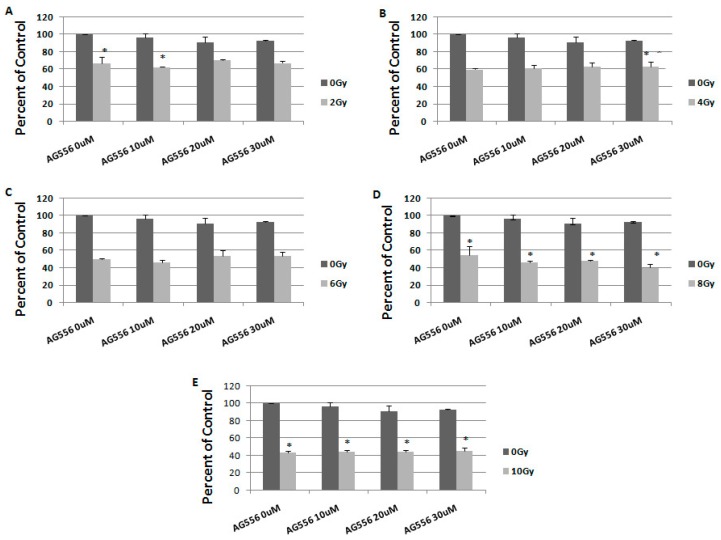
The effect of EGFR inhibition on ionizing radiation response in 11 HGG cells, seven days after the treatment. The cells were seeded in 96-well culture plates (3000 cells/well) then irradiated with 2 Gy (**A**); 4 Gy (**B**); 6 Gy (**C**); 8 Gy (**D**); 10 Gy (**E**) and treated with 10 µM, 20 µM or 30 µM AG556. The cells were incubated for seven days and cell viability was determined by MTT assay. All results show the mean of two independent experiments ± SD, * *p* < 0.05 vs. irradiated cells.

To study the long-term effect of the treatment, we evaluated the response to combination therapy, seven days after treatment. In 11 cells irradiated with 2 Gy, the treatment with 10 µM AG556 reduced cell survival by 4%, 20 µM AG556 treatment increased cell survival by 4% and the treatment with 30 µM AG556 increased cell survival by 1%, compared with radiation alone, one week after the treatment (Figure 7A).

The cytotoxic effect of 4 Gy gamma radiation was decreased by 2% with 10 µM AG556 treatment, by 4% with 20 µM AG556 treatment and by 4% with 30 µM AG556 treatment, seven days after the treatment (Figure 7B).

The cytotoxic effect of 6 Gy radiation was increased by 4% after the addition of 10 µM AG556 treatment, decreased by 3% with 20 µM AG556 treatment and also by 3% after exposure to 30 µM AG556 treatment, seven days after the treatment (Figure 7C).

The cytotoxic effect of 8 Gy radiation was increased by 8% after treatment with 10 µM AG556, by 6% with 20 µM AG556 treatment and by 14% when using 30 µM AG556 treatment, one week after the treatment (Figure 7D).

The cytotoxic effect of 10 Gy ionizing radiation was decreased by 1% after the addition of 10 µM AG556 treatment, by 1% with 20 µM AG556 treatment and by 2% with 30 µM AG556 treatment, seven days after the treatment (Figure 7E).

**Figure 8:**
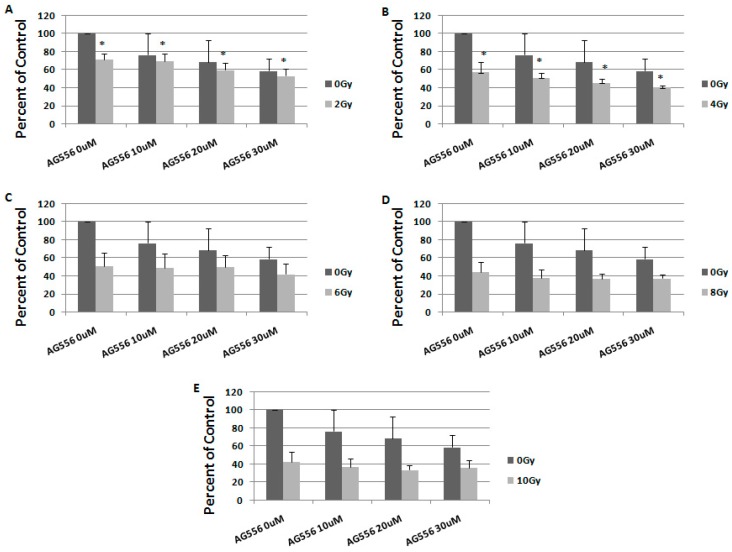
The effect of EGFR inhibition on ionizing radiation response in 15 HGG cells, seven days after the treatment. The cells were seeded in 96-well culture plates (3000 cells/well) then irradiated with 2 Gy (**A**); 4 Gy (**B**); 6 Gy (**C**); 8 Gy (**D**); 10 Gy (**E**) and treated with 10 µM, 20 µM or 30 µM AG556. The cells were incubated for seven days and cell viability was determined by MTT assay. All results show the mean of two independent experiments ± SD, * *p* < 0.05 vs. irradiated cells.

In 15 cells irradiated with 2 Gy, the treatment with 10 µM AG556 reduced cell survival by 9%, 20 µM AG556 treatment reduced cell survival by 19% and the treatment with 30 µM AG556 reduced cell survival by 25%, compared with radiation alone, one week after the treatment (Figure 8A).

The cytotoxic effect of 4 Gy gamma radiation was increased by 6% when using 10 µM AG556 treatment, by 12% after using 20 µM AG556 treatment and by 16% after using 30 µM AG556 treatment, seven days after the treatment (Figure 8B).

The cytotoxic effect of 6 Gy radiation was increased by 2% after 10 µM AG556 treatment, by 2% with 20 µM AG556 treatment and by 9% after treatment with 30 µM AG556, seven days after the treatment (Figure 8C).

The cytotoxic effect of 8 Gy radiation was increased by 6% after using 10 µM AG556 treatment, by 8% with 20 µM AG556 treatment and by 7% after exposure to 30 µM AG556 treatment, one week after the treatment (Figure 8D).

The cytotoxic effect of 10 Gy ionizing radiation was increased by 5% with 10 µM AG556 treatment, by 9% when exposed to 20 µM AG556 treatment and by 6% when treated with 30 µM AG556, seven days after the treatment (Figure 8E).

### 2.5. The Interaction between Combined Treatment with AG556 and Ionizing Radiation in 11 HGG Cells

The concomitant treatment with two or more therapeutic compounds is a common practice in clinical settings and the interaction between them can lead to subadditive, additive or synergistic effects. Of certain interest are those combinations in which two different approaches yield an additive or synergistic effect when they used together. As seen in Table 1, three days after treating the 11 HGG cells with the combined treatment we observed that 53% of the combinations were synergic, 40% were additive and only 7% were subadditive (Table 1).

In 15 HGG cells, 72 h after administration of the combined treatment 13% of the combinations were additive while 86% were subadditive (Table 2).

Seven days after the treatment of 11 HGG cells with the combined treatment, we observed that only one combination was synergic between 30 μm AG556 and 8 Gy radiation dose, 60% of combinations were additive and 33% of the combinations were subadditive (Table 3).

In 15 HGG cells, one week after the administration of the combined treatment, we observed that only 13% of combinations were additive while the rest of the combinations resulted in a subadditive interaction (Table 4).

## 3. Discussion

Radiotherapy remains one of the mainstays for brain tumours. However, in the case of HGGs, which are considered incurable neoplasms, resistance to radiation therapy is still a major clinical problem.

In our previously studies, we found that HGG cells respond differentially to ionizing radiation, two of three HGG cell lines studied being resistant, while one HGG cell line was sensitive to ionizing radiation [35,36].

The present study quantifies the three and seven-days effects of ionizing radiation with different single doses, on HGG cell lines 11 and 15 that displayed similar growth patterns. Our data showed a slight difference in the response to radiation of the HGG cell lines, with HGG line 15 presenting a slightly improved response when compared to HGG cell line 11 after three days, while seven days after the treatment the response presented by both cell lines was similar, with the exception of the 10 Gy radiation dose, which produced a stronger cytotoxic effect in the 11 HGG cell line.

In general, the cell lines were resistant to ionizing radiation, the highest dose of radiation used in this study (10 Gy) inducing 12% cell death in 11 cells and 26% cell death in 15 cells, three days after the radiation. However, this difference was no longer observed at the time extension after irradiation. Seven days after treatment, the cell lines proved to be resistant to the 2 Gy dose of ionizing radiation, but the increase in the radiation dose produced a substantial decrease in cell viability in both cell lines, reaching up to 66% cell death in 11 cells and 59% cell death in 15 cells.

Apart from the DNA’s self-repair capacity, many mitogenic signaling molecules are reported to be involved in tumour response to radiotherapy. Several studies have demonstrated that radiation alone triggers receptor tyrosine kinase activation and their common signaling pathways, resulting in radioresistance [37,38,39,40]. The combination of tumour specific agents with radiotherapy, is part of a pivotal effort to increase the sensitivity of the tumor cells response to radiation. Many receptor tyrosine kinases (RTKs) are overexpressed or overactivated in gliomas, making them suitable candidates for targeting agents in radio sensitization [41].

Among all these receptors, the EGFR is commonly mutated in GBMs, with the EGFRvIII receptor mutation being present in more than one third of tumors [42,43,44].

Thus, the current study was guided by the hypothesis that EGFR is a suitable target in HGGs. A retrospective study of EGFR prognostic value in GBM patients showed EGFR amplification and EGFRvIII expression in 40 patients and EGFR overexpression 39 patients of the 87 patients investigated. The authors suggested that EGFRvIII overexpression in the presence of EGFR amplification is a robust indicator of a poor survival prognosis [11].

Although EGFR expression was found to promote tumorigenicity and to be a strong indicator for a poor survival prognosis in GBM patients, inactivation of this mutant receptor by cetuximab (a recombinant monoclonal antibody) failed to produce significant clinical benefits in GBM patients [45]. Several clinical and preclinical studies reported an association between EGFR overexpression and radioresistance [46]. In accordance with these studies, we also found that 15 cells were slightly more sensitive to gamma radiation after three days when compared to 11 HGG cells, which expressed a low level of EGFR at the cell surface.

In the present study, an EGFR small molecule inhibitor (AG556) was used in combination with different ionizing radiation doses in single fractions, for radio sensitization of EGFR-expressing human HGG cells in vitro.

We found EGFR expression in two HGG cell lines. Like other studies published in this field, we have observed that the amount of EGFR on the cell surface is correlated with the level of cytotoxicity induced by receptor inactivation [47]. Thus, EGFR inactivation by AG556 was more cytotoxic in 15 HGG cells that expressed higher levels of EGFR at the cell surface, compared to 11 HGG cells. Many other studies also found that target inhibition of a specific RTK is inefficient in malignant glioma treatment, due to signal transduction redundancy and tumor heterogeneity [45]. The ability to disrupt the communication between the common signaling pathways emerging from several receptors is of paramount importance in the optimal development RTKs targeted therapies. In our previous studies, we found that dual targeting of PI3K/Akt/mTOR pathways was more efficient in killing GBMs cells than individual PDGFR or VEGFR inactivation [44,45]. The 11 HGG cell line was previously studied in terms of the intracellular signal of the receptor tyrosine kinases and was reported to have a ligand-independent Akt phosphorylation, which was connected to an increase in resistance to insulin-like growth factor-1 receptor inhibition [31]. Although, in line 15, the cytoxic effect of the AG556 was greater than in line 11, it should be noted that the efficacy of this small-molecule EGFR inhibitor as monotherapy in both cell lines has been modest, at best. Thus, the highest concentration of AG556 (30 μM) induced about 17% cell death, three days after the treatment and prolonged treatment time did not induce a more significant cytotoxicity in the 11 cell line. In 15 cells, the cytotoxicity induced by 30 μM AG556 was 33% at 3 days of treatment and 44%, seven days after the treatment.

Multitargeted tyrosine kinase inhibitors or combinations of these inhibitors with conventional therapy may be a potential way to exceed cancer cell resistance to single-agent treatment [36,48]. In a study by Vincenzo Damiano et al., a dual inhibition of VEGFR and EGFR signaling by ZD6474, increased the ionizing radiation effect of GBM, in vivo and in vitro. In this study, the authors found that GBMs cells were also sensitive to ZD6474 and the combination between this agent and radiotherapy synergized to induce an antitumor effects in vitro and in vivo treatment models [48].

We previously showed that dual targeting PDGFR and IGF1R was efficient in killing HGG cells [36,49]. However, the combination therapy of RTKs inhibitors and ionizing radiation, showed only a modest improvement in HGG cell cytotoxicity [35].

Regarding EGFR inhibition, we only analyzed the effect of receptor inactivation combined with TMZ, in one GBM cell line and found that this combination did not result in a greater therapeutic response when compared to the single treatment [50].

Here, we analyzed the effect of EGFR inhibition on radiation response in two HGG cell lines. We found that in the 11 HGG cell line, the combined treatment induced radiosensitivity in 93% of all combinations, three days after treatment. Extending treatment to seven days would have been expected to have a stronger radiosensitization effect on cells but, unexpectedly, the results showed that this effect decreased, the combined treatment inducing radio sensitivity in 67% of the analyzed combinations. In the 15 HGG cell line, the combined treatment of AG556 inhibitor with ionizing radiation failed to exert a superior cytotoxic effect in comparison to monotherapy, producing a subbaditive effect in 93% of the cases, 3 days after the treatment, and in 87% of the cases, seven days after the treatment. The molecular complexity that underlies radio-resistance may be the cause of the difference in the response of the cell lines to combined treatment. The resistance displayed by the cell lines after seven days can be attributed to several mechanisms. Malignant cells have the propensity to adapt and to overcome therapy by developing resistance. In addition, neoplastic cells can synthesize constitutive active receptors, making their inactivation by targeted therapy ineffective, over a long period of time.

In conclusion, our study showed that the cell lines responded differently to ionizing radiation, to EGFR inhibition and to combined treatment, despite their similar origin. To our knowledge, this is the first time the cumulative effect of the inhibitor AG556 and external gamma radiation was quantified, on malignant glioma cell lines. Various factors such as genomic instability, intrinsic radio-resistance, interactions between tumors and their micromedium, mitogenic signaling pathways could be used in development of targeted therapies and to adapt treatment approaches to individual patients.

## 4. Materials and Methods

### 4.1. Reagents

Anti-EGFR and anti-actin rabbit polyclonal IgG antibodies were from Santa Cruz; anti-rabbit and anti-mouse IgG horseradish peroxidase-linked antibodies and the ECl immunodetection reagents were purchased from Amersham Bioscience (Birmingham, UK); normal goat serum was purchased from Dako (Glostrup, Denmark), goat-anti-rabbit fluorescein isothiocyanate FITC was purchased from Jackson ImmunoResearch Laboratories, Inc. (JIR). (West Grove, PA, USA); 4′,6-diamidino-2-phenylindole (DAPI), Tyrphostine AG556 were purchased from Sigma (St. Louis, MO, USA). AG556 was diluted in dimethyl sulfoxide (DMSO) to a stock concentration of 10 mM and stored at 20 °C. The DMSO concentration was below 0.1% when the inhibitors were added in the cultured medium. MTT Cell Proliferation Kit was purchased from Roche Diagnostics GmbH (Mannheim, Germany).

### 4.2. Cell Cultures and Treatment

The primary cell cultures 11 and 15 HGG used in this study were established from tumours at the Academic University Hospital in Uppsala according to standard procedures. The cell lines have been previously characterized [31,32]. The cell lines were cultured in Mininum Essential Medium (MEM) containing 10% fetal bovine serum (FBS), 2 mM glutamine and antibiotic (100 IU/mL penicillin and 100 IU/mL streptomycin). The cells were grown in tissue culture flasks maintained in a 95% air/5% CO_2_ atmosphere at 37 °C in a humidified incubator. EGFR, were inhibited by 10, 20 and 30 μM AG556, for periods of times indicated in the figure legends. Appropriate control groups with diluents only were included.

### 4.3. Growth Kinetic Assay and Calculation of Cell Population Doubling Time

Cells were seeded in 12-well culture plates (2–5 × 10^4^ cells/well) and incubated at 37 °C in a humidified atmosphere of 5% CO_2_ overnight to adhere and then maintained in standard culture medium. During 7 days, one dish was trypsinized every day, and uniform cell suspension was counted in a Bürker hemocytometer (Sigma, St. Louis, MO, USA), using trypan blue. Cell viability was calculated as the percent of control. Each experiment was repeated three times. DT was calculated using the slope of the logarithmic phase of growth curve [51].

### 4.4. Irradiation

Cells were incubated in a 95% air/5% CO_2_ atmosphere at 37 °C in a humidified incubator in culture medium until 70–80% confluence. Cells were then irradiated in a culture medium at room temperature with 2, 4, 6, 8 and 10 Gy, using a 137Cs radiation source. The ionizing radiation was given in a single-dose and the cell proliferation was analyzed after 3 and 7 days, using the MTT assay.

### 4.5. MTT Assay

The cell proliferation was evaluated by MTT assay (Roche Diagnostic GmbH, Basel, Switzerland) that is based upon the cleavage of the yellow tetrazolium salt MTT to purple formazan crystals by metabolically active cells. 1 × 10 cells/w/200 μL medium were seeded in 96-well culture plates, incubated for 8 h and exposed to different concentrations of Tyrphostin. After 3 and 7 days of incubation, 10 μL of MTT labeling reagent were added to each well and plates were incubated at 37 °C for 4 h. Formazan products were solubilized and the optical density (OD) was measured at 595 nm. Results and relative cell viability were expressed as percentage of that in untreated control cultures.

### 4.6. Trypan Blue Exclusion Test

Cells were seeded in 12-well culture plates (2–5 × 10^4^ cells/well) and incubated at 37 °C in a humidified atmosphere of 5% CO_2_ overnight, to adhere. Cells were then treated with 10, 20 and 30 μM AG556 or irradiated in a culture medium at room temperature with 2, 4, 6, 8 and 10 Gy, using a 137Cs radiation source. After 3 and 7 days, the cells were harvested from the culture dishes, using trypsine and uniform cell suspension was counted in a Bürker hemocytometer, using trypan blue staining to determine the number of surviving and dead cells. Cell viability was calculated as the percent of control. Each experiment was repeated three times.

### 4.7. Flow Cytometry

The human HGG cells were detached, washed with fluorescence-activated cell counting (FACS) buffer (PBS containing 3% FBS and 0.02% NaN_3_), blocked in 10% FBS and incubated with anti-EGFR at working dilution 1:10 for 40–45 min at 4 °C. The cells were then stained with FITC-conjugated second antibody for 30 min on ice. IgG2 isotype control Ab was added for each conditions. Cells were analyzed using a FACS Calibur flow cytometer (BD Biosciences, Heidelberg, Germany) and the CellQuest^TM^ 3.3 software (San Jose, CA, USA). For each measurement, 100,000 events were acquired.

### 4.8. Western Blotting

Cells were lysed in lysis buffer (150 mM NaCl, 20 mM Tris–HCl (pH 7.5), 1% NP40, 1 mM EDTA, 1 mM EGTA, 1 mM sodium orthovanadate, and protease inhibitor mixture (Boehringer Mannheim Co., Barcelona, Spain) at 4 °C. The lysate was centrifuged (12,000× *g*) for 10 min at 4 °C to remove insoluble components. Protein was quantized by the BioRad Dc protein assay. Equal amounts of protein were separated on SDS-PAGE 10% gels and transferred to an Immobilon-P polyvinylidene fluoride (PVDF) membrane (Merck, Kenilworth, NJ, USA). The membrane was blocked with 5% nonfat dry milk in Tris-buffered saline containing 0.1% Tween 20 (TBST), then incubated with primary antibody in 1% nonfat dry milk in TBST, followed by a secondary antibody linked to horseradish peroxidase diluted in 1% nonfat dry milk in TBST. The ECL detection system for Western blot analysis was followed according to the manufacturer’s instructions for antibody detection.

### 4.9. Statistical Analysis

Statistical comparison of mean values was performed using the Student’s *t*-test. Differences with a *p*-value of *p* < 0.05 were considered statistically significant.

Interactions between drugs and radiation (I) were classified by the Multiplicative Method as follows: additive inhibition occurs when I1, 2 = I1 + I2; synergism occurs when I1, 2 > I1 + I2; and antagonism occurs when I1, 2 < I1 + I2 [52].

## Figures and Tables

**Table 1:** The interaction between combined treatment in 11 HGG cells, three days after the treatment.

Rad (Gy)	AG556 (µM)	Predicted Survival	Observed Survival	Effect
2	10	0.9	0.8	SYN
	20	0.8	0.8	ADD
	30	0.8	0.8	ADD
4	10	0.9	0.8	SYN
	20	0.9	0.8	SYN
	30	0.8	0.8	ADD
6	10	0.9	0.8	SYN
	20	0.8	0.8	ADD
	30	0.8	0.7	SYN
8	10	0.8	0.8	ADD
	20	0.8	0.7	SYN
	30	0.8	0.6	SYN
10	10	0.8	0.9	SUB
	20	0.8	0.8	ADD
	30	0.8	0.6	SYN

**Table 2:** The interaction between combined treatment in 15 HGG cells, three days after the treatment.

Rad (Gy)	AG556 (µM)	Predicted Survival	Observed Survival	Effect
2	10	0.7	0.7	ADD
	20	0.6	0.7	SUB
	30	0.6	0.6	ADD
4	10	0.6	0.7	SUB
	20	0.5	0.7	SUB
	30	0.5	0.7	SUB
6	10	0.5	0.7	SUB
	20	0.5	0.6	SUB
	30	0.5	0.6	SUB
8	10	0.5	0.7	SUB
	20	0.5	0.6	SUB
	30	0.5	0.6	SUB
10	10	0.6	0.7	SUB
	20	0.5	0.6	SUB
	30	0.5	0.6	SUB

**Table 3:** The interaction between combined treatment in 11 HGG cells, seven days after the treatment.

Rad (Gy)	AG556 (µM)	Predicted Survival	Observed Survival	Effect
2	10	0.6	0.6	ADD
	20	0.6	0.7	SUB
	30	0.6	0.7	SUB
4	10	0.6	0.6	ADD
	20	0.6	0.6	ADD
	30	0.6	0.6	ADD
6	10	0.5	0.5	ADD
	20	0.5	0.5	ADD
	30	0.5	0.5	ADD
8	10	0.5	0.5	ADD
	20	0.5	0.5	ADD
	30	0.5	0.40	SYN
10	10	0.4	0.5	SUB
	20	0.4	0.5	SUB
	30	0.4	0.5	SUB

**Table 4:** The interaction between combined treatment in 15 HGG cells, seven days after the treatment.

Rad (Gy)	AG556 (µM)	Predicted Survival	Observed Survival	Effect
2	10	0.6	0.7	SUB
	20	0.5	0.6	SUB
	30	0.5	0.5	ADD
4	10	0.4	0.5	SUB
	20	0.4	0.5	SUB
	30	0.3	0.4	SUB
6	10	0.4	0.5	SUB
	20	0.4	0.5	SUB
	30	0.3	0.4	SUB
8	10	0.4	0.4	ADD
	20	0.3	0.4	SUB
	30	0.3	0.4	SUB
10	10	0.3	0.4	SUB
	20	0.3	0.4	SUB
	30	0.3	0.4	SUB
